# Central Nervous System Myeloma and Unusual Extramedullary Localizations: Real Life Practical Guidance

**DOI:** 10.3389/fonc.2022.934240

**Published:** 2022-07-07

**Authors:** Vincenzo Sammartano, Alfonso Cerase, Valentina Venanzi, Maria Antonietta Mazzei, Beatrice Esposito Vangone, Francesco Gentili, Ivano Chiarotti, Monica Bocchia, Alessandro Gozzetti

**Affiliations:** ^1^ Hematology Unit, Department of Medicine, Surgery and Neuroscience, University of Siena, Azienda Ospedaliero Universitaria Senese, Siena, Italy; ^2^ Neuroimaging (Diagnostic and Functional Neuroradiology) Unit, Azienda ospedaliero-universitaria Senese, Siena, Italy; ^3^ Department of Medicine, Surgery and Neuroscience, University of Siena and Department of Radiological Sciences, Unit of Diagnostic Imaging, Azienda Ospedaliera Universitaria Senese, Siena, Italy

**Keywords:** multiple myeloma, extramedullary, CNS, neuro-imaging, therapy

## Abstract

Central nervous system localization of multiple myeloma (CNS-MM) accounts for about 1% of all MM during disease course or even rarer at diagnosis. A difference in the origin, i.e., osteodural or primary dural vs leptomeningeal/intraparenchymal, seems to define two distinct types of intracranial myeloma, with different clinical behavior. CNS-MM may occur also as a presentation of MM. Treatment is still unsatisfactory and many treatments have been reported: chemotherapy, intrathecal therapy, and radiotherapy, with dismal prognosis. Other sites of myeloma localization could be also of interest and deserve description. Because of the rarity and aggressiveness of the disease clinicians are often doubtful on how to treat it since there is no general agreement. Moreover, recent drugs such as the anti CD38 monoclonal antibody, immunomodulatory drugs, and proteasome inhibitors have changed the treatment of patients with MM with a significant improvement in overall response and survival. The role of novel agents in CNS MM management and unusual presentations will be discussed as well as the potential role of other new immunomodulatory drugs and proteasome inhibitors that seem to cross the blood-brain barrier. The purpose of this review is to increase awareness of the clinical unusual presentation and neuroradiological findings, give practical diagnostic advice and treatment options algorithm.

## Introduction

Multiple myeloma (MM) is a hematological malignancy characterized by proliferation of clonal plasma cells (PCs) within the bone marrow. The diagnosis of MM requires the presence of one or more myeloma defining events in addition to evidence of either 10% or more clonal PCs on bone marrow examination or a biopsy-proven plasmacytoma (1). MM has seen great survival improvements in latest years, due to novel drugs that acts with different mechanisms of action (2–4). Extramedullary multiple myeloma (EMM) is characterized by the ability of a clone and/or subclone of PCs to grow outside of the bone marrow microenvironment (5). EMM is defined by the presence of extramedullary disease in a patient meeting the definition of MM and does not include solitary extramedullary plasmacytoma or solitary bone plasmacytoma, which are considered distinct diagnostic entity (6). It could be differentiated EMM-extraosseous (EM-E) from EMM-bone related (EM-B), as the outcome is worst for patients with EM-E compared with those with EM-B (7, 8). EM-E is defined as EMM involving soft tissue or viscera in extraosseous locations resulting from hematogenous spread, not contiguous to involved bone, while EM-B is described as extramedullary plasma cell mass arising from the underlying bone with extension to contiguous paraskeletal area or soft tissue *via* cortical disruption (1). Some authors do not consider EM-B in the definition of EMM (9–12). Plasma cell leukemia (PCL) can be considered an extreme variant of aggressive EMM characterized by rapid progression, drug resistance and short survival (13–15), nevertheless some authors reason that PCL is a well-defined pathologic entity and should be excluded from the EMM spectrum, although PCL fulfills the definition of EMM (10). Longer patient survival, due to more efficient drugs and the following development of resistance to these therapies may engenders clones of plasm cells with unpredictable pattern of relapse (16). Authors argue that these locations represent a sanctuary not successfully treated by stem cell transplantation (17). EMM is usually associated with shorter overall and progression free survival, and resistance to conventional therapies (16). These lesions may potentially involve any organ and consist either of plasma cells proliferation or amyloid deposition (18). EMM can be present either at initial diagnosis (primary EMM) or at relapse (secondary EMM) (19). At diagnosis, EMM is typically found in skin and soft tissues; at relapse, typical sites involved include liver, kidneys, lymph nodes, central nervous system (CNS), breast, pleura, and pericardium (5, 12, 19–22).

MM with CNS involvement (CNS-MM) is a rare form of EMM characterized by PC infiltration of the CNS, leptomeninges or cerebrospinal fluid (CSF) (23). CNS-MM should be distinguished from osteodural or primary dural multiple myeloma (OD-DMM), in which extramedullary tumor masses most frequently arise from bone lesions in the cranial skull and rarer from primary dural involvement (24). Indeed, CNS involvement mostly occurs at relapse and patients often have had several lines of treatment (25). Since CNS-MM represents a minority of MM cases, available data come from single cases and retrospective studies (26). Few clinical series have been reported and CNS-MM is still difficult to manage (27). This may also result from the nature of the blood-brain barrier (BBB), that represents a natural protection from several drugs that are commonly used for the treatment of MM (28).

### Epidemiology

The reported incidence of EMM is generally underestimated (6), approximately 0.5-4.8% of MM cases at the time of diagnosis and 3.4-14% of MM cases in the advanced or relapse stage (5). Report incidence has recently increased (29), possibly in part due to improved survival as EMM represents the natural evolution of MM (30) and due to the increased use of PET/CT imaging as the recent International Myeloma Working Group guidelines recommend the use of PET/CT for both newly diagnosed and relapsed/refractory MM to determine the extent of bone damage and extramedullary involvement (31).

CNS-MM is diagnosed in less than 1% of MM patients with an overall survival reported less than 7 months (32–37). The reported median age of onset of CNS-MM is often younger than the usual of classical MM diagnosis; however, age at presentation varies between studies, suggesting CNS-MM may be underdiagnosed in older patients (24, 32, 38).

### Pathogenesis

Pathogenesis of extramedullary spread in multiple myeloma seems to be mediated by downregulation of chemokine receptors, adhesion molecules like CD56, and tetraspanins and upregulation of tumor promoter heparanase enzyme and CXCR4 (29, 39, 40). The PCAT-1/Wnt β-catenin signaling axis has also been implicated in EMM (41, 42). Several genetic features have been linked to extramedullary involvement in MM, including high-risk cytogenetics like t(4;14), t(14;16), gain(1q21), and del(17p) (43–45). Other possible risk factors include p53 deletion (46), CD56 downregulation (47–49), MAFB overexpression (12), and MYC overexpression (50). High levels of LDH have also been associated with EMM (51).

### Diagnosis

#### EMM and Unusual Localizations

Diagnosis of EMM is confirmed using imaging and/or by direct tissue sampling (40). PET/CT is the whole-body imaging technique of choice to detect EMM (52–54) and should be done in patients in whom extramedullary involvement is suspected based on clinical symptoms or considered at high risk for EMM (40). Biopsy of EMM lesion is useful especially in nonsecretory cases with no marrow involvement (6). Hess et al. analyzed 850 patients with MM and found 8 patients with atypical localizations of the disease: peritoneal dissemination, pulmonary amyloidosis with both infiltrative and nodular appearance, calcified mass of pulmonary hilum, nodular anaplastic plasmocytoma of the lung, multiple focal liver lesions, large soft-tissue mass of duodenum and large lesion involving nasopharynx (55). Also Patlas et al. found eight patients with EMM from a review of the radiological files of 200 myeloma patients with the following locations: breast, lymph nodes, thyroid cartilage, pancreas and stomach, adrenal and pleura, and meninges (56). According to multiple autopsy series, liver is the most frequent abdominal involved organ, both in the form of diffuse infiltration and focal hypovascularized lesions (57). Kidney are rarely involved whereas retroperitoneal perirenal masses resembling lymphoma are described (58). Adrenal involvement is rare and consists of heterogeneous soft tissue lesions (59). Pancreatic involvement present most commonly as solitary lesion or multiple lesions with avid arterial enhancement, mimicking neuroendocrine tumors (60). Lymph node involvement is also described and the most common sites are paratracheal, parasplenic and supraclavicular. Lymph modes appear enlarged or conglomerate (57). Regarding gastrointestinal localizations, the most frequent is small bowel followed by stomach, colon and esophagus (61). Lesions appear as large masses with mural infiltration, mimicking lymphoma (16). Testicular and ovarian involvement is quite rare, presenting as diffuse enlargement or solid mass (62). Myeloma deposits may finally occur in subcutaneous tissue or muscles as nodules or diffusely infiltrating lesions (60). CT and PET/CT are optimal to localize EMM outside the CNS (6).

#### CNS-MM

**Figure 1:**
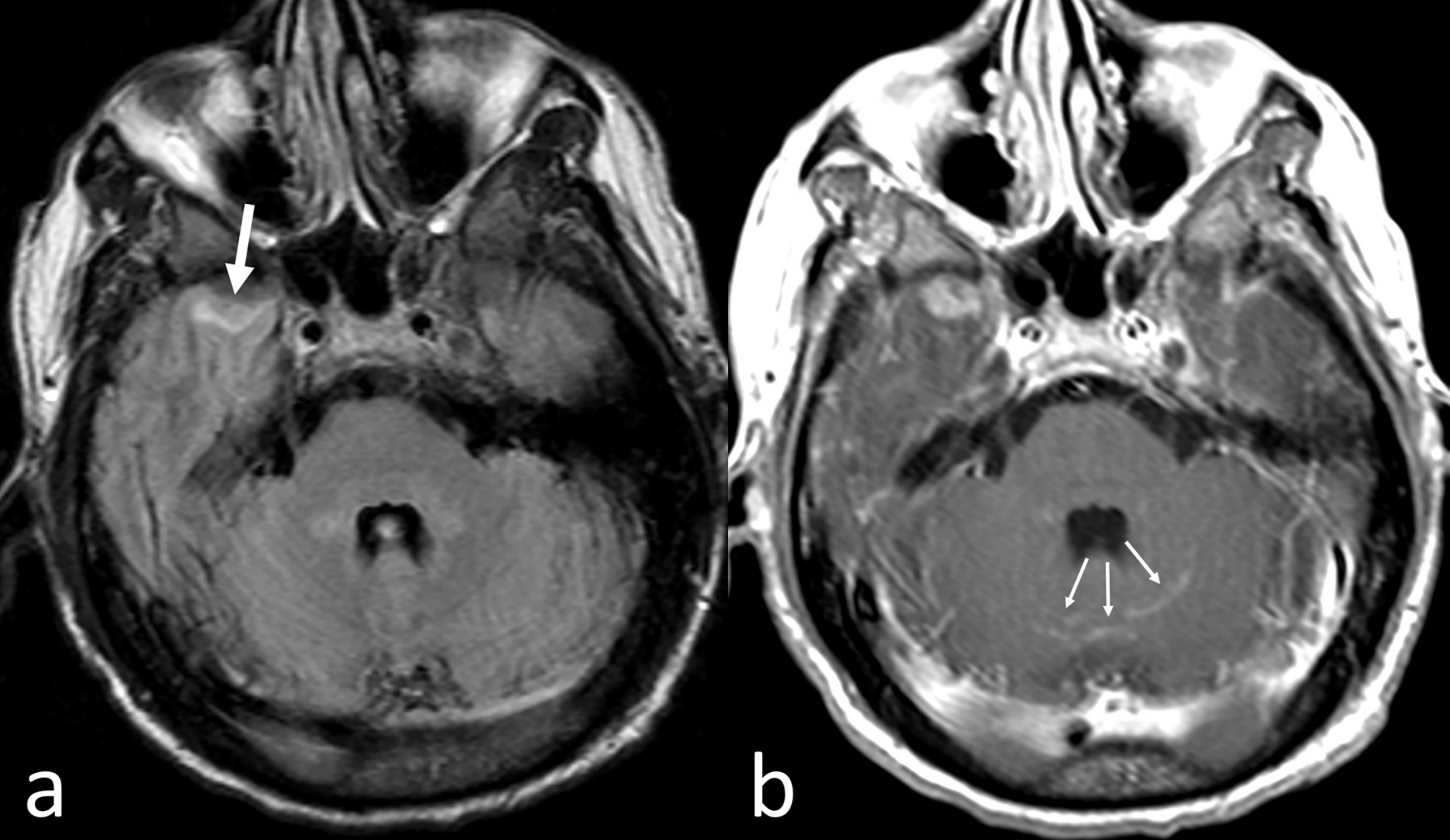
CNS-MM. Despite artifactual images from patient’s movement, fast fluid-attenuated inversion recovery axial MR image **(A)** shows a small area of abnormal intensity in the surface of the anterior pole of the right temporal lobe (thick arrow). Corresponding Gadolinium-enhanced T1-weighted axial MR image **(B)** shows enhancement not only of the left temporal lesion but also in some cerebellar sulci (thin arrows).

CNS-MM can be diagnosed by the presence of monoclonal immunoprotein or atypical plasma cells in the CSF by conventional cytology and flowcytometry, imaging evidence of intraparenchymal lesions or leptomeningeal/dural enhancement and/or direct tissue sampling, especially in doubtful cases (23, 63, 64). Diagnosis of CNS-MM still represents a challenge as neurological symptoms may be confounded and attributed to other clinical situations, such as hyper viscosity, hypercalcemia, uremia, paraproteinemia, bone damage, treatment-related neuropathy, stroke or opportunistic infections (28). Indeed, CNS-MM can produce heterogeneous symptoms, including impairments to sight, speech, motor and sensory functions, headache, radicular pain, confusion, dizziness and, less frequently, seizures, vomiting, cranial nerve palsy, lethargy and convulsion (32, 65). If unexplained neurological findings occur at any time during the disease course in patients with MM, then either MRI or a CSF examination should be promptly performed (23–66). MRI with gadolinium contrast has a superior sensitivity than cranial CT to assess for CNS-MM (6) (**Figure 1**). Imaging is effective in most cases, although it is associated with a false negative rate of 10%, thus, it is preferable to perform imaging, pathological, and CSF examination concurrently (32).

Detection of plasma cells in CSF provides strong evidence of CNS-MM, although these can be absent in patients with parenchymal infiltration or isolated changes in the dura mater (32, 67). Cytological techniques can detect atypical plasma cells and flow cytometry can detect monoclonal CD38/CD138 expressing cells in CSF in approximately 90% of CNS-MM cases (32, 68). CSF cytology may also be useful to identify unknown tumors, whereas flow cytometry may distinguish the clonal plasma cells found in MM from polyclonal plasma cells present in CSF in other conditions (69, 70).

Furthermore, the presence of a paraprotein, including clonal free light chains (FLC), in CSF can be diagnostic and the parallel evidence of minute or undetectable concentrations of paraprotein in the serum may represent that monoclonal immunoprotein detected in CSF originates from plasma cells in the CNS rather than BM (23).

### Therapy

#### EMM

Available data regarding treatment are derived almost entirely from retrospective studies.

Regimens containing bortezomib and/or IMiDs have improved outcomes in patients with EMM; however, the gains in PFS and OS are less pronounced compared with classic MM (7, 12, 20, 21, 46, 49, 51, 71–81). In a recent meta-analysis, patients with EMM (91% EM-B) treated with IMiDs, mainly lenalidomide, or bortezomib had PFS like multiple myeloma patients (72). Few studies suggest that ASCT can overcome the poor prognostic impact of EMM, whereas in most studies the benefit of ASCT in patients with EMM appears to be more limited (7, 12, 20, 21, 46, 50, 51, 71, 74, 76, 77). Poor outcome after single ASCT can be attributed to high-risk cytogenetics which can be found in almost 40% patients with EMM (20). Upfront tandem transplant has been shown to overcome poor outcomes in these patients compared to single ASCT (82), however the EBMT Registry has recently reported no benefit of tandem ASCT over single ASCT; thus, the superiority of tandem ASCT cannot be convincingly established (20).

Patients with EMM have a higher risk of relapse, even after transplant (44). In relapsed/refractory patients with EMM, lymphoma-like polychemotherapy regimen such as PACE, Dexa-BEAM, and HyperCVAD followed by ASCT or auto-allo-SCT have shown efficacy (83, 84). In patients responding to rescue therapy, allo-SCT should be considered as a platform for additional therapeutic strategies to take advantage of the graft versus myeloma effect (29).

Regarding radiotherapy (RT), there is no consensus on its use in EMM, but few cases reported good outcomes in patients with EMM (22, 29, 85–88).

There are limited data regarding the efficacy of daratumumab, an anti-CD38 monoclonal antibody approved for treatment of newly diagnosed and relapsed MM, in EMM (89). An updated pooled analysis of studies (GEN501 part 2 and SIRIUS) evaluating the role of daratumumab in heavily pre-treated patients reported an overall response rate of 16.7% in a subset of patients with EMM (90). The European Myeloma Network (EMN) is conducting a phase II trial of daratumumab in association with bortezomib, cyclophosphamide and dexamethasone in patients with MM and extramedullary disease (EMN19 study, NCT 04166565).

Novel agents such as isatuximab, selinexor and melflufen might be effective in EMM, but data are limited (91–93).

BCMA chimeric antigen receptor T cells (CAR-T) have shown promising results in a limited number of relapsed patients with EMM and in a meta-analysis on BCMA CAR-T cell therapy, the presence of EMM at time of infusion was not associated with lower response rates (94, 95).

#### CNS-MM

No standard treatment for CNS-MM has yet been established and the current approach includes systemic therapy, intrathecal (IT) therapy, and CNS irradiation, often in combination (23). Main clinical studies are reported in **Table 1**.

**Table 1:** Reported case series on CNS myeloma in the literature.

Author	Year	N	MM-CNS dx	FISH-high risk	PI-IMIDs/ASCT	OD	OS (months)	Parenchimal/meningis	OS (months)
**Gozzetti et al., 2012 (Italy)** (24)	‘00-’12	50	14	47%	70%/20%	38	25	12	6
**Jurczyszyn et al., 2016** **(Internat. 20 centers)** (32, 88)	‘00-’15	173	25	35%	57%/NR	NO	NA	103	7
**Varga et al., 2018 (Hungary)** (26)	‘07-’17	13	0	61.5%	46%/7.6%	NA	NA	NA	4
**Paludo et al., 2016 (US, Mayo)** (33)	‘98-’14	29	10	72%	44%/0%	NO	NA	29	5
**Katodritou et al., 2015 (Greece)** (96)	‘00-’13	31	29	NR	60%/0.5%	24	11	7	3
**Chen et al., 2013** **(CAN, Toronto)** (38)	‘99-’10	37	15	NR	70%/5%	NO	NA	37	5
**Dias et al., 2017 (Brazil)** (66)	‘08-’16	20	16	NA	10%/0%	19	NR	3	5.8
**Badros et al., 2016 (US)** (101)	‘08-’16	2	0	100%	100%/0%	NO	NA	2	NR
**Abdallah et al., 2014** **(US, Little Rock)** (37)	NR	35	15	45%	NR/42%	NO	NA	35	4
**Gangatharan et al., 2012** **(Australia)** (34)	‘01-’10	7	24	NR	NR	NO	NA	7	2
**Lee et al., 2013 (Australia)** (36)	‘00-’11	17	36	NR	41%/0%	NR	NR	11	4

MM-CNS dx= diagnosi of CNS myeloma; FISH-high risk= del17p, t4;14,t14;16; PI-IMIDs/ASCT= proteasome inhibitors-immunomodulatory drugs/autologous stem cell tranplantation.

Systemic therapy successfully employed in MM might be ineffective in CNS-MM due to: tumor resistance after previous therapy because they require interaction with the BM microenvironment, or due to the inability to cross the BBB (32, 65, 97). For this reason, it is important to choose agents that have the potential to cross the BBB. In a large retrospective study, the only group to have a significantly longer median OS than the untreated group received systemic treatment (OS 12 vs. 3 months), thus highlighting the importance of systemic therapy (32).

Standard chemotherapy lacks efficacy in CNS-MM as alkylating agents including melphalan and cyclophosphamide are poor at penetrating the BBB, while high-dose methotrexate or cytarabine are ineffective against myeloma. Bendamustine can cross the BBB and has shown some efficacy in two cases of leptomeningeal relapse of myeloma in combination with thalidomide, dexamethasone and craniospinal irradiation (98). High-dose steroids are known to penetrate the BBB, but they have limited efficacy as monotherapy.

Proteasome inhibitors (PI) in regular clinical use (bortezomib, carfilzomib and ixazomib) cannot penetrate the BBB (99); however, bortezomib has been reported to enhance radiosensitivity and chemosensitivity when used in combination with other agents in CNS-MM probably due to pathological changes such as inflammation and angiogenesis increasing the permeability of the BBB (24, 100). Marizomib, a newer irreversible PI, has shown to distribute uniformly within the brain parenchyma and has proved potential efficacy in relapsed refractory MM and a small number of CNS-MM patients, thus making it a suitable agent to be tested in clinical trials (101).

Immunomodulatory drugs (IMiDs) have demonstrated the capability to cross the BBB. Indeed, cases of successful treatment with thalidomide or lenalidomide have been reported and pomalidomide has demonstrated activity in EMD and good penetrance of the BBB (34, 35, 96, 98, 102–107).

The usual intrathecal therapy (IT) regimen administered in CNS-MM is the triplet of IT hydrocortisone, methotrexate and/or cytarabine, which is repeated until clearance of plasma cells and free light chains from the CSF. However, studies have only shown a modest benefit of IT therapy and its use is controversial as myeloma cells are not thought to be particularly susceptible to methotrexate or cytarabine and it is unlikely to penetrate parenchymal CNS lesions (36, 38, 108).

Malignant plasma cells are highly radiosensitive (109); thus, radiotherapy is effective for CNS involvement especially when combined with systemic therapy (24). Although whole brain radiation is a therapeutic option in CNS-MM, its practical application is limited due to toxicity. There is increasing evidence that modern radiotherapy techniques can deliver optimal responses in CNS-MM without significant myelotoxicity (110).

The role of ASCT is unclear, but it is thought to be able to overcome the poor prognosis of CNS-MM (111, 112). The longest survivor (25 months) in a study of 18 CNS-MM patients had received an allo-SCT after the diagnosis of CNS-MM, suggesting a graft-versus-myeloma effect in the CNS (35).

Daratumumab can be measurable in CSF, demonstrating the capability to cross the BBB (113). Indeed, daratumumab has been reported to be effective in CNS-MM in combination with IT therapy and/or radiotherapy (26, 114).

Novel agents like isatuximab, elotuzumab and venetoclax have shown efficacy in MM and are worth exploring in the CNS-MM subset. Immunotherapy modalities targeting the BCMA (CAR-T, BiTE and ADC) might also have a role in CNS-MM, but there are still no data in this setting.

### Real Life Approach

EMM represents an aggressive form of MM characterized by poor prognosis and should be managed as an ultra-high-risk disease. PET/CT is the gold standard to detect EMM in the body and should be performed in clinical practice for all patients with a suspicion of EMM, such as those with clinical symptoms, high LDH serum levels or revised stage III. When neurological symptoms develop, it is necessary to undergo head CT and/or MRI, and further investigate for CNS involvement by a CSF examination. Direct tissue sampling is not always indicated, but it should be performed in unclear cases.

Treatment approach should be adapted to patient age and fitness and, if possible, patients should be considered for enrollment in clinical trials. Younger/fit patients with bulky EMM should receive intensive induction therapy with regimens incorporating a proteasome inhibitor and alkylators, such as V (or K)-RD-PACE to provide rapid reduction in disease burden. In cases with less bulky disease and for patients with organ dysfunction or significant comorbidities, frontline therapy with a triplet regimen that includes a proteasome inhibitor and an IMiD, such as RVd or KRd is the preferred option. Patients failing to achieve a partial response after two cycles, if eligible, should be timely switched to a more aggressive regimen. Radiation therapy may be employed in selected cases as palliative. Daratumumab could be added in the upfront therapy in transplant ineligible patients. In the elderly population and in frail patients, the risk of life-threatening adverse events should be carefully evaluated and dose reductions or a palliative approach could be offered upfront. After induction therapy, in responding transplant-eligible patients, an upfront ASCT with high dose melphalan is recommended to achieve a deeper response, then maintenance therapy should be offered to obtain likely longer disease control. Based on limited data, a tandem ASCT could be considered, whereas an allo-SCT may be performed preferably only in the setting of a clinical trial. Transplant ineligible patients usually continue induction regimen for several cycles followed by extended maintenance, preferably with a combination of a proteasome inhibitor and an IMiD.

At relapse, suggested treatments are based on lymphoma-like regimens such as PACE or Dexa-BEAM, although response rate is about 50% and duration of response is typically ≤ 4 months. In patients who are eligible, auto or allo-SCT could be considered, provided that the patient is in response at the time of transplant. Beyond this, novel-agent combinations (e.g., carfilzomib-, isatuximab-, melflufen-, selinexor-based) or immunotherapy, if available, may be considered.

For CNS-MM, a backbone of systemic therapy incorporating IMiD and high-dose steroid, intrathecal and radiation therapy appears to provide the best treatment outcome. If available, daratumumab should be added to systemic therapy as promising results have been reported.

MM response criteria, including MRD assessment, should be applied in patients with EMM, in addition PET/CT and/or MRI should be done at three months after treatment initiation and at physician discretion thereafter. To declare complete remission (CR), all evidence of EMM must have disappeared.

## Conclusion

Despite prognosis of patients with MM has recently improved with the advent of new therapies, EMM and CNS-MM still carry poor outcomes. Clinical data derive from retrospective studies and case reports; indeed, prospective trials are lacking due to the rarity of the diseases. However, ongoing MM trials are employing sensitive imaging techniques, thus information regarding EMM and CNS-MM might be extrapolated. International randomized multi-center studies are warranted to better understand the risk factors, the biological and genetic features and to assess the efficacy and safety of available treatment options and the impact of novel therapeutic agents. Hence, solid data and guidelines could be generated to further improve outcomes in EMM and CNS-MM.

## Author Contributions

AG, VS designed the study and wrote the manuscript. AC, VV, BV wrote the manuscript. MM wrote the manuscript. MB revised the manuscript. All authors approved the final version of the paper. MB and AG share co-last authorship.

## Conflict of Interest

The authors declare that the research was conducted in the absence of any commercial or financial relationships that could be construed as a potential conflict of interest.

## Publisher’s Note

All claims expressed in this article are solely those of the authors and do not necessarily represent those of their affiliated organizations, or those of the publisher, the editors and the reviewers. Any product that may be evaluated in this article, or claim that may be made by its manufacturer, is not guaranteed or endorsed by the publisher.
